# Correction to: Treatment comparison of femoral shaft with femoral neck fracture: a meta-analysis

**DOI:** 10.1186/s13018-020-01892-3

**Published:** 2020-08-20

**Authors:** Yao Lu, Yakang Wang, Zhe Song, Qian Wang, Liang Sun, Cheng Ren, Hanzhong Xue, Zhong Li, Kun Zhang, Dingjun Hao, Yang Zhao, Teng Ma

**Affiliations:** Department of Orthopaedic Surgery, HongHui Hospital, Xi’an Jiaotong University, Xi’an, 710054 Shaan’xi Province China

**Correction to: Journal of Orthopaedic Surgery and Research 15, 19 (2020)**

**https://doi.org/10.1186/s13018-019-1496-z**

Following publication of the original article [1], we have been informed that references from Table 1 did not match the list. This Correction provides the corrected table with the missing references.

**Table 1:** Characteristic of the included studies

Study	Year	Language	Country	Age range (mean)	Groups	*n*	Years of onset
Akgul [25]	2016	English	Turkey	17.6 ± 1.8	Reconstruction nail	5	September 2007 to June 2013
					Hollow screw+plate	10	
Boese [26]	2016	English	Germany	18.2 ± 2.1	Reconstruction nail	8	October 2008 to June 2010
					Hollow screw+plate	9	
Genest [27]	2018	English	Germany	54.7 ± 12.1	Reconstruction nail	15	June 2010 to July 2016
					Hollow screw+plate	15	
Jiang [28]	2015	English	China	62.4 ± 18.7	Reconstruction nail	233	January 2009 to October 2014
					Hollow screw+plate	233	
Kovala k [29]	2017	English	Turkey	74.1 ± 4.1	Reconstruction nail	13	January 2009 to January 2015
					Hollow screw+plate	18	
Maranho [20]	2018	English	America	22.3 ± 1.7	Reconstruction nail	53	May 2000 to March 2014
					Hollow screw+plate	49	
Oh [21]	2017	English	Japan	78.2 ± 7	Reconstruction nail	10	August 2015 to February 2017
					Hollow screw+plate	11	
Ripamonti [22]	2014	English	Italy	68.4 ± 9.5	Reconstruction nail	38	April 2000 to March 2010
					Hollow screw+plate	166	
Sangeux [23]	2015	English	Australia	56.7 ± 2.3	Reconstruction nail	11	February 2002 to June 2010
					Hollow screw+plate	11	
Yamauchi [24]	2016	English	Japan	72.1 ± 11.2	Reconstruction nail	101	January 2010 to January 2012
					Hollow screw+plate	99	

Correct:

[25]. Akgül, T., et al., Double intertrochanteric osteotomy for trochanteric overgrowth and a short femoral neck in adolescents. Journal of orthopaedic surgery (Hong Kong), 2016. 24(3): p. 387–391.

[26]. Boese, C.K., et al., The Modified Femoral Neck-Shaft Angle: Age- and Sex-Dependent Reference Values and Reliability Analysis. BioMed Research International, 2016. 2016: p. 1–7.

[27]. Genest, F. and L. Seefried, Subtrochanteric and diaphyseal femoral fractures in hypophosphatasia—not atypical at all. Osteoporosis International, 2018. 29(8): p. 1815–1825.

[28]. Jiang, N., et al., Femoral Version, Neck-Shaft Angle, and Acetabular Anteversion in Chinese Han Population. Medicine, 2015. 94(21): p. e891.

[29]. Emrah Kovalaka, C.E.T.A., Management of unstable pertrochanteric fractures with proximal femoral locking compression plates and affect of neck-shaft angle on functional outcomes. Journal of Clinical Orthopaedics and Trauma, 2017(421): p. 1–6.
